# Ultralow Oxygen Tension (2%) Is Beneficial for Blastocyst Formation of In Vitro Human Low-Quality Embryo Culture

**DOI:** 10.1155/2022/9603185

**Published:** 2022-06-01

**Authors:** Mingzhao Li, Xia Xue, Juanzi Shi

**Affiliations:** The ART Center, Northwest Women's and Children's Hospital, Xi'an, China

## Abstract

**Objectives:**

To investigate whether a reduction in the O_2_ tension from 5 to 2% during extended culture from day 3 onwards was beneficial to human blastocyst development in vitro.

**Methods:**

We firstly identified 139 patients who had no low-quality embryos on day 3, and all the embryos were prolonged to culture on day 5 or 6. We mainly analyzed 188 patients receiving IVF/ICSI-ET for the first time, and no high-quality embryos were obtained on day 3 from January 2018 to December 2019. After transferred with one or two low-quality embryos, extended culture was performed under low O_2_ (5%) or ultralow O_2_ (2%) tension for surplus embryos. 296 embryos from 106 patients were continued to culture under 5% O_2_ tension, and 214 embryos from 82 patients were continued to culture under 2% O_2_ tension. Main outcomes compared were blastulation and high-quality blastulation rates.

**Results:**

We observed no significant differences in the blastulation and high-quality blastulation rates for high-quality cleavage-stage embryos between 2% and 5% O_2_ groups (*p* > 0.05). For low-quality cleavage-stage embryos, we observed that the 2% O_2_ group showed a significantly higher blastulation (39.72 versus 31.08%; *p* = 0.043) rate than that in the 5% O_2_ group. The high-quality blastocyst formation rate (10.75 versus 8.45%; *p* = 0.380) was comparable between the 2% and 5% O_2_ groups. The blastulation rate reached 44.86% by culturing blastocysts an additional day under 2% O_2_ tension which was significantly higher than that (32.09%) under 5% O_2_ tension (*p* < 0.05).

**Conclusion:**

A reduction in O_2_ tension from 5 to 2% after day 3 might be beneficial to the patients with no high-quality embryos. Extended culture to day 7 under 2% O_2_ tension increased the number of available blastocysts per IVF/ICSI cycle and was worth recommending especially for patients with few blastocysts.

## 1. Introduction

Culture of human embryos was traditionally performed under atmospheric O_2_ tension of about 20%. In 1971, the scientists noticed the first efficient culture of a human embryo from day 2 to day 5 and emphasized that the O_2_ in the culture system was not about 20% (atmospheric O_2_), but that the gas phase was 5% O_2_ (physiologic O_2_), 5% carbon dioxide, and 90% nitrogen [1]. During this period, 20% and 5% O_2_ were widely used in human embryo culture and showed similar developmental rates.

Multiple meta-analysis reviews demonstrated an increase in pregnancy and live birth rates at 5% O_2_ embryo culture compared with that at 20% O_2_ [2, 3]. Human embryo culture under 2-8% O_2_ was recommended by ESHRE revised guidelines for good practice in IVF labs. Thus, 5% O_2_ embryo culture was widely adopted in most IVF labs.

It was generally accepted that O_2_ tension was lower in the uterus than in the oviduct, and that the embryo crossed the uterotubal junction sometime on day 3. In 2017, Morin proposed that the optimal O_2_ tension in embryo culture might depend on the stage of development [4]. Meanwhile, Kaser put forward the hypothesis which assumed that sequential O_2_ exposure (5% from days 1 to 3, then 2% from days 3 to 5) would improve blastocyst yield and quality compared to continuous exposure to 5% O_2_ among human preimplantation embryos [5]. Nevertheless, Munck et al. showed a reduction in O_2_ tension from 5 to 2% O_2_ after day 3 did not improve embryo development, quality, and utilization rate [6]. Therefore, it was controversial whether 2% O_2_ tension was more suitable than 5% O_2_ for extended culture of human embryos.

Embryonic genome activation always occurred at the 8-cell stage; so, it appeared that blastocyt-stage embryo transfer (BSET) had a higher success rate than cleavage-stage embryo transfer (CSET). Although the utilization of BSET had made as more IVF centers switched from day-3 cleavage stage to routine day-5/6 transfers of blastocyst-stage embryos, there were a majority of patients who had no opportunity to try blastocyst transfers especially for the patients with no high-quality embryos on day 3. It was confirmed that multifetal pregnancies carried high risks of poor clinical outcomes, and it could be effectively avoided by the adoption of elective single BSET [7]. Thus, it was significant to make some improvements in blastocyst formation and blastocyst quality by some technical advancements and revised professional guidance.

In this study, we aimed to investigate whether a reduction in O_2_ tension from 5 to 2% during extended culture from day 3 onwards was beneficial to human blastocyst development in vitro.

## 2. Materials and Methods

### 2.1. Patients

This study included 139 patients who had no low-quality embryos and 188 patients who had no high-quality embryos on day 3 from January 2018 to December 2019. All the embryos were prolonged to culture on day 5 or 6 under low O_2_ (5%) or ultralow O_2_ (2%) tension for the patients with no low-quality embryos. After transferred with one or two low-quality embryos, extended culture was performed under 5% or 2% O_2_ tension for surplus embryos of the patients with no high-quality embryos. Main outcomes compared were blastulation and high-quality blastulation rates. All female patients were not older than 38 years to eliminate possible age-related cycle characteristics, and all cycles were the first attempted downregulated ovarian stimulation cycles.

The study protocol was approved by the Ethics Committee of Northwest Women and Children's Hospital (No. 2022007). Patient consent was not required due to the retrospective nature of the study.

### 2.2. Ovarian Stimulation Protocol

The ovarian stimulation protocol was described previously [8]. In brief, stimulation protocols were used with a combination of GnRH agonist/GnRH antagonist and recombinant FSH. The ovarian response was monitored by serial ultrasound examination and hormone measurement. Ten thousand units of human chorionic gonadotrophin (hCG) were administered to patients when three follicles were >18 mm. Oocyte retrieval was performed 36 h later by transvaginal ultrasonography-guided aspiration.

### 2.3. Embryo Culture and Assessment

The OCCs were cultured in the medium (IVF; Vitrolife, Sweden) after retrieval. Fertilization was performed 39 to 40 hours (39-42 hours for ICSI) after HCG administration while incubated in fertilization medium (IVF; Vitrolife). The zygotes were shifted to cleavage medium (G-1; Vitrolife) 5 hours after IVF fertilization. The embryos to culture blastocysts on day 3 were transferred to blastocyst medium (G-2; Vitrolife) until day 6. All media were covered with paraffin oil in a humidified atmosphere at 37°C for prior 24 h.

The same brands of incubators (Model c200; Labotect, Germany) were used for both 2% and 5% O_2_ tension to culture embryos. The 5% O_2_ tension was to culture in 6% CO_2_, 5% O_2_, and 89% N_2_. The 2% O_2_ culture condition also had 6% CO_2_ in air. All other conditions were equal except O_2_ tension between the two systems. In our center, we had two IVF labs with equal conditions. After transferred with one or two low-quality embryos, extended culture was performed under 2% O_2_ in one lab and 5% O_2_ in the other lab.

The cleavage-stage embryo scoring system used a combination of blastomere number, homogeneous degree of blastomeres, and degree of fragmentation. In this study, the low-quality cleavage-stage embryo should meet the following criteria: (i) 4-5 blastomeres, even homogeneous blastomeres < 10%cytoplasmic fragmentation; (ii) 6-7 blastomeres, even homogeneous blastomeres with nearly 15% cytoplasmic fragmentation or one uneven blastomere < 5%cytoplasmic fragmentation; (iii) 8-10 blastomeres, even homogeneous blastomeres with nearly 20% cytoplasmic fragmentation or one uneven blastomere < 15%cytoplasmic fragmentation or two uneven blastomeres < 5%cytoplasmic fragmentation; and (iv) >10 blastomeres, even homogeneous blastomeres with nearly 10% cytoplasmic fragmentation or one uneven blastomere < 5%cytoplasmic fragmentation.

The scoring system for blastocyst evaluation was a combination of the stage of development from 1 to 6 (early, blastocyst, full blastocyst, expanded, hatching/hatched) and of the grade of the inner cell mass (ICM; A, tightly packed, many cells; B, loosely grouped, several cells; or C, very few cells.) and of the trophectoderm (TE; A, many cells forming a cohesive epithelium; B, few cells forming a loose epithelium; or C, very few large cells.) [9]. In this study, the blastocyst scored ≥3BB was defined as high-quality blastocyst.

### 2.4. Statistical Analysis

Comparisons of the results between groups in the case of continuous variables were analyzed by using Student's *t*-test for data with normal distribution and nonparametric Mann–Whitney *U*-test for data with skewed distribution. Comparisons of the results between groups in the case of categorical variables were expressed as number and percentage and analyzed using the chi-square test or Fisher's exact test. The statistical analysis was performed with SPSS version 21 (IBM Corp., USA). Differences were considered statistically significant when *p* < 0.05.

## 3. Results

### 3.1. Demographic and Baseline Characteristics

**Figure 1:**
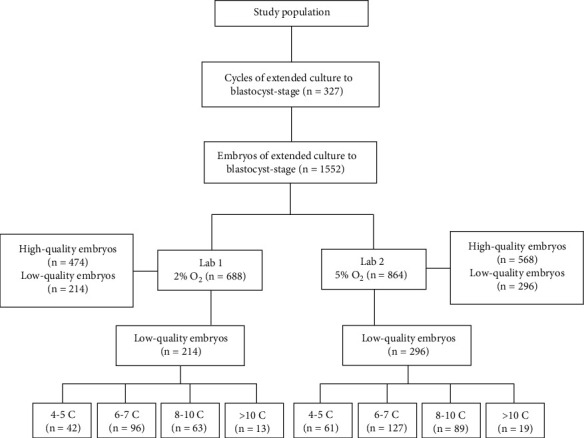
Study flow chart. 4-5 C: embryos with 4-5 blastomeres; 6-7 C: embryos with 6-7 blastomeres; 8-10 C: embryos with 8-10 blastomeres; >10 C: Embryos with more than 10 blastomeres.

The study flow chart was shown in Figure 1. A total of 1552 cleavage-stage embryos were prolonged for extended culture to blastocyst stage. 474 high-quality embryos from 63 patients were cultured under 2% O_2_ tension, and 568 high-quality embryos from 76 patients were cultured under 5% O_2_ tension. A total of 188 patients who had no high-quality embryos on day 3 underwent IVF/ICSI-ET during the study period were included. After transferred with one or two low-quality embryos, extended culture was performed under 5% or 2% O_2_ tension for surplus embryos. A total of 296 embryos from 106 patients were cultured under 5% O_2_ tension, and 214 embryos from 82 patients were cultured under 2% O_2_ tension. The embryos for extended culture to blastocyst stage were divided into four subgroups according to the different numbers of blastomeres as follows: 4-5 C (embryos with 4-5 blastomeres), 6-7 C, 8-10 C, and >10 C groups. The demographic and baseline characteristics of the study population were described in Tables 1 and 2, no significant differences were observed in the female age, BMI, basal FSH value, basal *E*_2_ value, Gn stimulation time, total Gn dose, the number of retrieved oocytes, infertile time, and proportion of IVF/ICSI (*p* > 0.05).

### 3.2. Development of High-Quality and Low-Quality Cleavage-Stage Embryos

We observed no significant differences in the blastulation (71.10 versus 70.07%; *p* = 0.717) and high-quality blastulation (54.64 versus 54.75%; *p* = 0.971) rates between the 2% and 5% O_2_ groups (Table 3). In the 2% O_2_ group, the proportion of embryos with 4-5 C, 6-7 C, 8-10 C, and >10 C was 19.63%, 44.86%, 29.44%, and 6.07%, respectively. In the 5% O_2_ group, the proportion of embryos with 4-5 C, 6-7 C, 8-10 C, and >10 C was 20.61%, 42.91%, 30.07%, and 6.42%, respectively. No significant differences were observed in the proportion of day 3 low-quality embryos with different blastomeres between 2% and 5% O_2_ groups (*p* > 0.05) (Table 4). The embryos cultured under 2% O_2_ tension showed a significantly higher blastulation rate than those cultured under 5% O_2_ tension (39.72 versus 31.08%; *p* = 0.043). The blastulation rate was improved with no significant difference under 2% O_2_ tension compared with 5% O_2_ tension for the low-quality embryos with 4-5 C (26.19 versus 22.95%; *p* = 0.706), 6-7 C (36.46 versus 29.92%; *p* = 0.303), 8-10 C (50.79 versus 35.96%; *p* = 0.068), and >10 C (53.85 versus 42.11%; *p* = 0.513). The high-quality blastulation rate was comparable either under 2% O_2_ tension or 5% O_2_ tension (10.75 versus 8.45%; *p* = 0.380) (Table 5).

### 3.3. Blastocyst Outcomes of Extended Culture with Low-Quality Cleavage-Stage Embryos to Day 7

**Figure 2:**
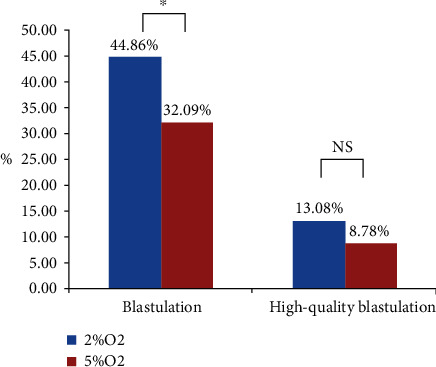
Blastocyst development of extended culture with low-quality cleavage-stage embryos to day 7 between the 2% and 5% O_2_ groups. ^∗^ was significantly different; NS: no significant differences.

We observed that 11 embryos and 3 embryos achieved blastulation on day 7 under 2% and 5% O_2_ tension, respectively. The blastulation and high-quality blastulation rates were improved from 39.72% and 10.75% to 44.86% and 13.08% under 2% O_2_ tension, respectively. The blastulation and high-quality blastulation rates showed no obvious improvement from 31.08% and 8.45% to 32.09% and 8.78% under 5% O_2_ tension, respectively (Figure 2).

## 4. Discussion

O_2_ tension was lower in the uterus than in the oviduct, and the human intrauterine O_2_ tension had been measured to be around 2% when the human embryo reached the uterine cavity on day 3.5. Kaser et al. analyzed the blastocyst development of normally and abnormally fertilized embryos under 2 or 5% O_2_ tension from day 3 onwards. Culture under 2% O_2_ tension led to a higher blastulation rate (40.2 versus 22.5%) and a higher utilization rate (36.8 versus 21.3%) [10]. A recent study showed that the total and usable blastocyst rates (44.4 versus 54.8% and 21.8 versus 32.8%, respectively) were significantly higher under 2% O_2_ tension from day 3 onwards [11].

For patients with no high-quality embryos, cleavage-stage embryo transfer was always recommended in cases of blastocyst formation failure. It was difficult to select an optimal embryo from several low-quality embryos by morphology evaluation for the embryologists. Extended culture of low-quality cleavage-stage embryos might be an alternative strategy. In this study, we mainly aimed to explore whether a reduction in the O_2_ tension from 5 to 2% during from day 3 onwards was beneficial for the blastocyst development of low-quality embryos.

Our results indicated that a reduction in O_2_ tension from 5 to 2% after day 3 did not improve the blastulation rate and blastocyst quality of high-quality cleavage-stage embryos. It was significant that we observed that embryo culture in biphasic (5-2%) O_2_ concentration significantly increased the total blastocyst formation rate of low-quality cleavage-stage embryos compared with culture in monophasic (5%) O_2_ concentration. This suggested that mimicking the physiological O_2_ concentrations was beneficial to the blastocyst development of low-quality cleavage-stage embryos.

It was well known that the blastulation rate was associated with the number of blastomeres. Kong et al. showed that the blastocyst formation rate and high-quality blastocyst formation rate increased with cell number and reached high levels in the 7-8 C and >10 C groups [12]. Luna et al. observed that human blastocyst morphological quality was significantly improved in embryos classified as fast on day 3 (>or = 10 cells) [13]. In this study, both the 2% and 5% O_2_ groups showed a similar proportion of day 3 low-quality embryos with different blastomeres. Embryos with a similar number of blastomeres were cocultured to the blastocyst stage to identify from which blastocyst was formed. Our results showed that a reduction in the O_2_ tension from 5 to 2% during from day 3 onwards improved the blastulation rate beyond 10% for the embryos with ≥8 blastomeres; although, no significant differences were observed. It suggested that the problem of slow embryonic development might not be improved by extended culture under ultralow O_2_ tension.

Another important factor affecting embryo quality was the homogeneous degree of blastomeres. The embryos with uneven cell cleavage had a lower developmental capacity in comparison with evenly cleaved embryos, and uneven cleavage might result in embryos with a higher degree of aneuploidy and/or multinuclear rate [14]. The problem of uneven cell cleavage might not be improved by ultralow O_2_ tension. We suspected that the extended culture under ultralow O_2_ tension was most likely to improve the blastulation rate of low-quality embryos with normal blastomere number and more cytoplasmic fragmentation. Embryo cytoplasmic fragmentation was associated with oxidative stress in embryos, and the presence of fragmentation was harmful to the subsequent development of embryos due to the loss of cytoplasmic mitochondria, mRNA, and regulatory proteins [15]. Kim et al. showed that early fragment removal on in vitro fertilization day 2 significantly improved the subsequent development and clinical outcomes of fragmented human embryos [16]. Fragmentation removal was needed to a laser hole in the zona pellucida and used a hand-made micropipette with a mouthpiece to suction out the fragments from around and between the blastomeres. The ultralow O_2_ tension culture seemed safer for fragmentation removal in comparison with manual removal.

High-quality cleavage-stage embryos showed a higher opportunity of excellent blastocyst formation compared to day 3 low-quality embryos [17]. For low-quality cleavage-stage embryos, it should be less likely to form high-quality blastocysts. Our results showed a similar high-quality blastulation rate between 2% and 5% O_2_ groups. Thus, the ultralow O_2_ tension culture could improve the blastulation rate but not the high-quality blastulation rate. It was demonstrated that the blastocyst from the low-quality embryo had a comparable implantation rate [18]. Therefore, it might be helpful for such patients with bad prognosis to obtain more blastocysts.

Selection of available blastocysts typically occurred on days 5/6, and some embryos with slow development formed blastocysts on day 7. Should extended culture to blastocyst stage include day 7? Whitney et al. indicated that extended culture to day 7 was beneficial by achieving viable euploid embryos that would have otherwise been discarded [19]. A review concluded that culturing embryos an additional day increased the number of useable embryos per cycle and provided further opportunities for pregnancy for patients, especially those who had only a few or low-quality blastocysts [20]. In this study, we observed that the blastulation rate of extended culture with low-quality embryos to day 7 showed better improvement under 2% O_2_ tension than 5% O_2_ tension. Thus, it might be more worthy of consideration to culture blastocysts to day 7 if no blastocysts were obtained on day 5/6 for patients with no high-quality embryos on day 3.

In conclusion, extended culture of low-quality cleavage-stage embryos under 2% O_2_ tension might be an alternative strategy for patients with no high-quality embryos on day 3. There was no doubt that a prospective study was needed to further confirm our findings.

## Figures and Tables

**Table 1:** Main characteristics of the patients with no low-quality embryos on day 3.

Parameter	2% O_2_	5% O_2_	*P*
No. of cycles	63	76	/
Female age (y)	30.85 ± 3.16	31.02 ± 3.22	0.623
BMI (kg/m^2^)	21.57 ± 3.21	21.68 ± 3.28	0.702
Basal FSH (mIU/ml)	6.52 ± 1.53	6.63 ± 1.47	0.757
Basal *E*_2_ (pg/ml)	56.78 ± 30.92	55.82 ± 31.01	0.636
Gn stimulation time (d)	10.73 ± 2.05	10.65 ± 2.00	0.825
Total Gn does (IU)	27.09 ± 10.11	27.54 ± 10.05	0.629
No. of retrieved oocytes (*n*)	12.15 ± 3.51	11.86 ± 3.37	0.513
Infertile time (years)	3.01 ± 1.47	2.97 ± 1.39	0.801
Fertilization methods			0.448
IVF	77.78% (49/63)	82.89% (63/76)	
ICSI	22.22% (14/63)	17.11% (13/76)	

Continuous variables were presented as mean ± standard deviation. Categorical variables were expressed as number and percentage. BMI: body mass index; FSH: follicle-stimulating hormone; *E*_2_: estradiol; Gn: gonadotropin; IVF: in vitro fertilization; ICSI: intracytoplasmic sperm injection.

**Table 2:** Main characteristics of the patients with no high-quality embryos on day 3.

Parameter	2% O_2_	5% O_2_	*P*
No. of cycles	82	106	/
Female age (y)	31.23 ± 3.47	31.36 ± 3.52	0.533
BMI (kg/m^2^)	21.83 ± 3.41	22.06 ± 3.49	0.611
Basal FSH (mIU/ml)	6.71 ± 1.58	6.82 ± 1.61	0.781
Basal *E*_2_ (pg/ml)	52.33 ± 30.06	53.12 ± 31.29	0.629
Gn stimulation time (d)	10.82 ± 2.07	10.63 ± 2.01	0.810
Total Gn does (IU)	27.96 ± 10.13	27.68 ± 10.02	0.748
No. of retrieved oocytes (*n*)	10.05 ± 3.29	9.96 ± 3.17	0.607
Infertile time (years)	3.06 ± 1.51	3.25 ± 1.60	0.782
Fertilization methods			0.959
IVF	66 (80.49%)	85 (80.19%)	
ICSI	16 (19.51%)	21 (19.81%)	

**Table 3:** Blastocyst development of high-quality cleavage-stage embryos between the 2% and 5% O_2_ group.

Parameter	2% O_2_	5% O_2_	*P*
Extended culture to blastocyst stage (*n*)	474	568	/
Blastulation (%, *n*)	71.10% (337/474)	70.07% (398/568)	0.717
High-quality blastulation (%, *n*)	54.64% (259/474)	54.75% (311/568)	0.971

**Table 4:** Proportion of low-quality cleavage-stage embryos with different blastomeres between the 2% and 5% O_2_ groups.

Parameter	2% O_2_	5% O_2_	*P*
No. of embryos	214	296	
4-5 C (%, *n*)	19.63% (42/214)	20.61% (61/296)	0.785
6-7 C (%, *n*)	44.86% (96/214)	42.91% (127/296)	0.661
8-10 C (%, *n*)	29.44% (63/214)	30.07% (89/296)	0.878
>10 C (%, *n*)	6.07% (13/214)	6.42% (19/296)	0.874

**Table 5:** Blastocyst development of low-quality cleavage-stage embryos between the 2% and 5% O_2_ group.

Parameter	2% O_2_	5% O_2_	*P*
Extended culture to blastocyst stage (*n*)	214	296	/
Blastulation (%, *n*)	39.72% (85/214)^∗^	31.08% (92/296)^∗^	0.043
Blastulation with different blastomeres			
4-5 C (%, *n*)	26.19% (11/42)	22.95% (14/61)	0.706
6-7 C (%, *n*)	36.46% (35/96)	29.92% (38/127)	0.303
8-10 C (%, *n*)	50.79% (32/63)	35.96% (32/89)	0.068
>10 C (%, *n*)	53.85% (7/13)	42.11% (8/19)	0.513
High-quality blastulation (%, *n*)	10.75% (23/214)	8.45% (25/296)	0.380

^∗^ was significantly different.

## Data Availability

The data used to support the findings of this study were available from the corresponding author upon request.

## References

[1] Steptoe P. C., Edwards R. G. (1976). Reimplantation of a human embryo with subsequent tubal pregnancy. *Lancet*.

[2] Nastri C. O., Nóbrega B. N., Teixeira D. M. (2016). Low versus atmospheric oxygen tension for embryo culture in assisted reproduction: a systematic review and meta-analysis. *Fertility and Sterility*.

[3] Belli M., Antonouli S., Palmerini M. G. (2020). The effect of low and ultra-low oxygen tensions on mammalian embryo culture and development in experimental and clinical IVF. *Systems Biology in Reproductive Medicine*.

[4] Morin S. J. (2017). Oxygen tension in embryo culture: does a shift to 2% O_2_ in extended culture represent the most physiologic system?. *Journal Of Assisted Reproduction And Genetics*.

[5] Kaser D. J. (2017). On developing a thesis for Reproductive Endocrinology and Infertility fellowship: a case study of ultra-low (2%) oxygen tension for extended culture of human embryos. *Journal Of Assisted Reproduction And Genetics*.

[6] Munck N. D., Janssens R., Segers I., Tournaye H., Van de Velde H., Verheyen G. (2019). Influence of ultra-low oxygen (2%) tension on in-vitro human embryo development. *Human Reproduction*.

[7] Katler Q. S., Kawwass J. F., Hurst B. S. (2022). Vanquishing multiple pregnancy in in vitro fertilization in the United States-a 25-year endeavor. *American Journal of Obstetrics and Gynecology*.

[8] Shi W. H., Zhang S. L., Zhao W. Q. (2013). Factors related to clinical pregnancy after vitrified-warmed embryo transfer: a retrospective and multivariate logistic regression analysis of 2313 transfer cycles. *Human Reproduction*.

[9] Gardner D. K., Lane M., Stevens J., Schlenker T., Schoolcraft W. B. (2000). Blastocyst score affects implantation and pregnancy outcome: towards a single blastocyst transfer. *Fertility And Sterility*.

[10] Kaser D. J., Bogale B., Sarda V., Farland L. V., Williams P. L., Racowsky C. (2018). Randomized controlled trial of low (5%) versus ultralow (2%) oxygen for extended culture using bipronucleate and tripronucleate human preimplantation embryos. *Fertility And Sterility*.

[11] Brouillet S., Baron C., Barry F. (2021). Biphasic (5-2%) oxygen concentration strategy significantly improves the usable blastocyst and cumulative live birth rates in in vitro fertilization. *Scientific Reports*.

[12] Kong X., Yang S., Gong F. (2016). The relationship between cell number, division behavior and developmental potential of cleavage stage human embryos: a time-lapse study,. *PLoS One*.

[13] Luna M., Copperman A. B., Duke M., Ezcurra D., Sandler B., Barritt J. (2008). Human blastocyst morphological quality is significantly improved in embryos classified as fast on day 3 (>or=10 cells), bringing into question current embryological dogma. *Fertil Sterility*.

[14] Hardarson T., Hanson C., Sjögren A., Lundin K. (2001). Human embryos with unevenly sized blastomeres have lower pregnancy and implantation rates: indications for aneuploidy and multinucleation. *Human Reproduction*.

[15] Blerkom J. V., Davis P., Alexander S. (2001). A microscopic and biochemical study of fragmentation phenotypes in stage-appropriate human embryos. *Human Reproduction*.

[16] Kim S. G., Kim Y. Y., Park J. Y. (2018). Early fragment removal on in vitro fertilization day 2 significantly improves the subsequent development and clinical outcomes of fragmented human embryos. *Clinical and Experimental Reproductive Medicine*.

[17] Mullin C. M., Fino M. E., Talebian S., Krey L. C., Licciardi F., Grifo J. A. (2010). Comparison of pregnancy outcomes in elective single blastocyst transfer versus double blastocyst transfer stratified by age. *Fertility Sterility*.

[18] Kaartinen N., Das P., Kananen K., Huhtala H., Tinkanen H. (2015). Can repeated IVF-ICSI-cycles be avoided by using blastocysts developing from poor-quality cleavage-stage embryos?. *Reproductive Biomedicine Online*.

[19] Whitney J. B., Balloch K., Anderson R. E., Nugent N., Schiewe M. C. (2019). Day 7 blastocyst euploidy supports routine implementation for cycles using preimplantation genetic testing. *JBRA Assisted Reproduction*.

[20] Hammond E. R., Cree L. M., Morbeck D. E. (2018). Should extended blastocyst culture include day 7?. *Human Reproduction*.

